# Prognostic Value of Highly Expressed Type VII Collagen (COL7A1) in Patients With Gastric Cancer

**DOI:** 10.3389/pore.2021.1609860

**Published:** 2021-08-26

**Authors:** Sung Eun Oh, Mi Yun Oh, Ji Yeong An, Jun Ho Lee, Tae Sung Sohn, Jae Moon Bae, Min-Gew Choi, Kyoung-Mee Kim

**Affiliations:** ^1^ Department of Surgery, Samsung Medical Center, Sungkyunkwan University School of Medicine, Seoul, South Korea; ^2^ Department of Pathology and Translational Genomics, Samsung Medical Center, Sungkyunkwan University School of Medicine, Seoul, South Korea

**Keywords:** biomarker, prognosis, gastric cancer, tumor microenvironment, type VII collagen (COL7A1), distant metastasis

## Abstract

Collagen is a major component in the tumor microenvironment. This study reveals a novel biomarker candidate, type VII collagen (COL7A1), in patients with gastric cancer. To identify genes differentially expressed in gastric cancer tissue, we analyzed cancerous (*n* = 20) and noncancerous tissues (*n* = 13) using a DNA microarray. To perform immunohistochemistry and validate the upregulation of COL7A1 expression, we collected 200 more gastric cancer tissues and 100 normal gastric tissues from 200 randomly selected patients who underwent gastrectomy for gastric cancer between January 2010 and December 2013. The correlations between COL7A1 expression and clinicopathological parameters and patients’ overall survival (OS) were analyzed. In the microarray, COL7A1 was upregulated in gastric cancer tissue compared with normal tissue. In the immunohistochemistry study, COL7A1 was more highly expressed in cancer tissue than in normal tissue (*p* = 0.001). Patients with intracellular COL7A1 expression had significantly poorer five-year OS than those with only extracellular expression (41.5 versus 69.7%, *p* = 0.001), and the site of expression was an independent prognostic factor of OS (hazard ratio 2.00, 95% CI 1.26–3.16, *p* = 0.003). Also, we found a significant association between the COL7A1 immunohistochemistry score and distant metastasis (high versus low, odds ratio 4.45, 95% CI 1.40–14.16, *p* = 0.011). The site and total immunohistochemistry score of COL7A1 expression in gastric cancer showed prognostic significance for OS and distant metastasis, respectively. COL7A1 could be a novel biomarker with diagnostic and therapeutic value.

## Introduction

With the development of diagnostic techniques for early detection [1] and adjuvant treatments [2, 3], the long-term survival rate of gastric cancer patients in Korea improved between 2011 and 2015 [4]. However, gastric cancer remains one of the most common cancers in Korea, and it causes high cancer-related mortality worldwide [5]. To overcome its poor prognosis and the limitations of palliative treatment for unresectable, metastatic, or recurrent gastric cancer, researchers and clinical physicians are focusing on the tumor microenvironment in their search for novel biomarkers or a target therapy.

The tumor microenvironment is deeply connected to tumor progression and metastasis in gastric cancer [6]. Among the various components of the tumor microenvironment, collagen is a major structure in the extracellular matrix. Twenty different collagen types have been identified so far [7]; among them, the association with gastric cancer has been studied only for type I. Previous studies revealed that increases in the expression of type I correlate with poor clinical outcomes in gastric cancer patients [8, 9].

Unlike fibril-forming collagens (type I) and basement membrane collagens (type IV), type VII collagen (encoded by the COL7A1 gene) functions as the anchoring fibrils for basement membranes and is mostly distributed at the dermal-epidermal junction of skin, the oral mucosa, and the cervix, where it is covered with stratified squamous epithelium [7]. COL7A1 mutations cause an incurable, potentially fatal skin disease called dystrophic epidermolysis bullosa, which is characterized by chronic skin fragility and blistering [10]. Cutaneous squamous cell carcinoma shows an increase in aggressive behavior and metastatic potential when mutated COL7A1 loses its function [11]. In esophageal squamous cell carcinoma, COL7A1 expression correlates with the depth of tumor invasion, lymphatic invasion, and prognosis [12]. The clinicopathological significance of unregulated COL7A1 expression in gastric cancer has not yet been studied.

Our aim in this study was to reveal the clinical significance and prognostic effects of a newly found upregulation of COL7A1 expression in gastric cancer patients. First, we explored many genes differentially expressed in gastric cancer tissue, including COL7A1, with a DNA microarray. Second, we performed immunohistochemistry to evaluate the levels of COL7A1 expressed in gastric cancer tissue and adjacent normal tissue. Third, we further investigated the association between the clinicopathologic variables of gastric cancer patients and COL7A1 expression and evaluated the prognostic significance of COL7A1 expression levels.

## Methods

### Microarray Gene Analysis

A microarray gene analysis (HumanHT-12 v4 BeadChip, Illumina, CA, United States) was performed using gastric cancer (*n =* 20) and normal gastric tissues (*n =* 13). This array contains 47,231 probes designed to cover content from NCBI RefSeq Release 38 (November 7, 2009), as well as legacy UniGene content. Briefly, complementary DNA was synthesized from tissue RNA and directly hybridized to the microarray according to the manufacturer’s instructions (LAS Inc., Seoul, Korea). Signals from the prepared microarray were scanned, and we used the fluorescent intensities to generate data for the whole genes detected. We sifted the meaningful gene data by subtracting background signals. In addition, to show similarities in the expression of 4,509 genes among the 33 samples, hierarchical clustering and pairing of the gene expression profiles was performed. After the application of additional statistical criteria, we identified differentially expressed genes (DEGs). A functional annotation analysis of each gene list was performed using the functional annotation tool in the Database for Annotation, Visualization, and Integrated Discovery (DAVID) [13]. DAVID is a comprehensive set of functional annotation tools and has been used for the systematic and integrative analysis of large gene lists. For this work, we highlighted the most relevant terms from each functional annotation category in the Kyoto Encyclopedia of Genes and Genomes (KEGG) pathways and visualized the clustered DEGs in a heatmap plot using an in-house R script. We used the color key, which is log_10_ (fragments per kilobase of transcripts per million mapped reads, FPKM +1) values to compare gene expression across genes and samples.

### Patients and Samples for Immunohistochemistry

To validate the selected target gene as a novel biomarker, we collected cancerous (*n* = 200) and normal stomach tissues (*n* = 100) from 200 randomly selected advanced gastric cancer patients who received a gastrectomy at Samsung Medical Center between January 2010 and December 2013. The surgical specimens of those patients were stored at –180°C (in nitrogen) in the Biobank at Samsung Medical Center (Certified KS Q ISO 9001:2009).

The median follow-up for the patients (*n =* 200) was 53.0 months (range: 0–115). We reviewed the following clinicopathologic characteristics: age, sex, tumor size, tumor location, gross type, histologic type, histologic subtype, Lauren type, depth of invasion, number of metastatic lymph nodes, lymph node metastasis, distant metastasis, pathologic stage, lymphatic invasion, perineural invasion, and venous invasion. The histologic type was categorized as differentiated or undifferentiated. Well or moderately differentiated adenocarcinoma was categorized to the differentiated group. Poorly differentiated adenocarcinoma, signet ring cell, and mucinous type cancers were sorted to the undifferentiated group. The histologic subtype was further sorted into four categories: tubular, papillary, signet ring cell, and mucinous. The pathologic stage was classified according to the eighth edition of the American Joint Committee on Cancer Classification. We obtained survival data for the patients from their updated medical records and the National Statistical Office in South Korea. This study was approved by the Institutional Review Board of Samsung Medical Center, Seoul, Korea (SMC 2018-11-040).

### Immunohistochemistry Staining

Tissue was embedded in Tissue-Tek® OCT compound (Sakura, United States) and frozen in liquid nitrogen. For staining, 7 µm cryosections of each tissue were air-dried. After fixation with masked formalin (10% non-buffered fluid, DN-4301, DANA, Korea for 10 min) and permeabilization (0.1% Triton X-100 in peroxidase blocking solution for 15 min at room temperature (RT)), we applied 200 µL of peroxidase blocking solution per slide for 15 min at RT. After applying 200 µL per slide of 1:200 diluted primary antibody (Abnova, PAB19138, United States), we incubated the slides overnight at 4°C. Next, 200 µL of antibody enhancer per slide (Polink-2 Plus Rabbit HRP kits, GBI (Golden Bridge International), United States) was applied and incubated for 30 min at RT. After that, we applied 200 µL of polymer per slide (Polink-2 Plus Rabbit HRP kits, GBI, United States) and incubated them for another 30 min at RT. Finally, immunostaining was done with diaminobenzidine solution (DAKO, Agilent, United States). Between steps, the slides were washed three times for 5 min in PBS. The slides were then counterstained with hematoxylin (ScyTek, United States) and mounted.

### Grading of COL7A1 Expression

The final immunohistochemistry staining results were scored by one pathologist who was blinded to the clinicopathological characteristics of the patients. The scores were determined as the sum of the intensity and extent of staining, as suggested by a previous study [14]. Staining intensity was scored as 0 (negative), 1 (weakly positive), 2 (moderately positive), or 3 (strongly positive). The extent of staining was scored as the percentage of the area with positive staining: 0 (0–10%), 1 (11–30%), 2 (31–50%), 3 (51–70%), and 4 (71–100%). The tissue staining scores were determined as the sum of the score of the intracellular area (cancer cell or glandular cell; range: 0–7) and the extracellular area (stroma; range: 0–7). The tissue staining scores (range: 0–14) of type VII collagen expression were then divided into two groups for comparison and analysis: the high expression group (total score ≥ 8) and low expression group (total score < 8). We also categorized the patients into two groups by the area of staining: patients whose tissues were stained only at the extracellular matrix and patients whose tissues were also stained at the intracellular space. Because the staining was not even across each slide, the pathologist interpreted the intensity and extent by comparing each sample with negative control (definite: lymphocyte; relative: normal gastric epithelial cell, smooth muscles, nerve fibers) and positive control (fibrocollagenous stroma tissue, fibrohistiocytes) areas of each slide.

### Statistical Analysis

Gene data analysis software (GenomeStudio Gene Expression Module v1.6+, Illumina, CA, United States) was used in the microarray study. The scanned genes in all samples were prefiltered to eliminate genes with missing signals, signal intensity < 0, or a detection *p* value > 0.05. DEGs were selected using |fold change| ≥ 2 and *Q* value ≤ 0.005. Expression quantification and quantile normalization were performed before the DEG analysis. The Mann-Whitney test was used with significance set at *p <* 0.05, and the Benjamini-Hochberg test was used for multiple testing correction.

The two hundred patients were randomly selected for additional tissue collection through a statistics program. Because no previously reported criteria were available to define cut-off values for the immunohistochemistry scores, we used the Contal and O’Quigley method [15], which maximizes the log-rank statistic, to find the optimal cut-off values for the overall survival analysis. We used the cut-off value (= 8) to categorize patients into high and low score groups (*p* = 0.132). To investigate the clinicopathologic factors of the categorized patients, we used the χ^2^ or Fisher exact test for categorical variables and the Mann-Whitney test for continuous variables. Five-year overall survival (OS) was calculated using the Kaplan-Meier method. The log-rank test was used for the univariate analysis. A Cox proportional hazards model with the backward logistic regression method was used for the multivariate analysis of variables with a *p <* 0.05 in the univariate analysis. Last, we used a backward logistic regression to find statistically significant risk factors for distant metastasis. This model was evaluated using the area under the receiver operating characteristics curve (AUC) to measure its predictive accuracy. The statistical analyses, including the random selection of patients, were processed using SPSS version 25.0 for Windows (SPSS, Chicago, IL), SAS version 9.4 (SAS Institute, Cary, NC), and R 3.6.1 (Vienna, Austria; http://www.R-project.org/).

## Results

### Gene Analysis in Gastric Cancer Tissue

After initial scanning of the fluorescent signals, we found 34,602 genes in the tumor and normal tissues. After pairing and clustering cancerous and noncancerous tissues, the accumulated count of DEGs that survived filtering for technical reliability (no missing signals, signal intensity ≥ 0, and detection *p* value ≤ 0.05) and gradually elevating statistical probability (|fold change, FC| ≥ 2 and *Q* value ≤ 0.005) was 1,662. Among them, 918 genes were upregulated, and 744 genes were downregulated in tumor tissue compared with normal tissue.

**FIGURE 1:**
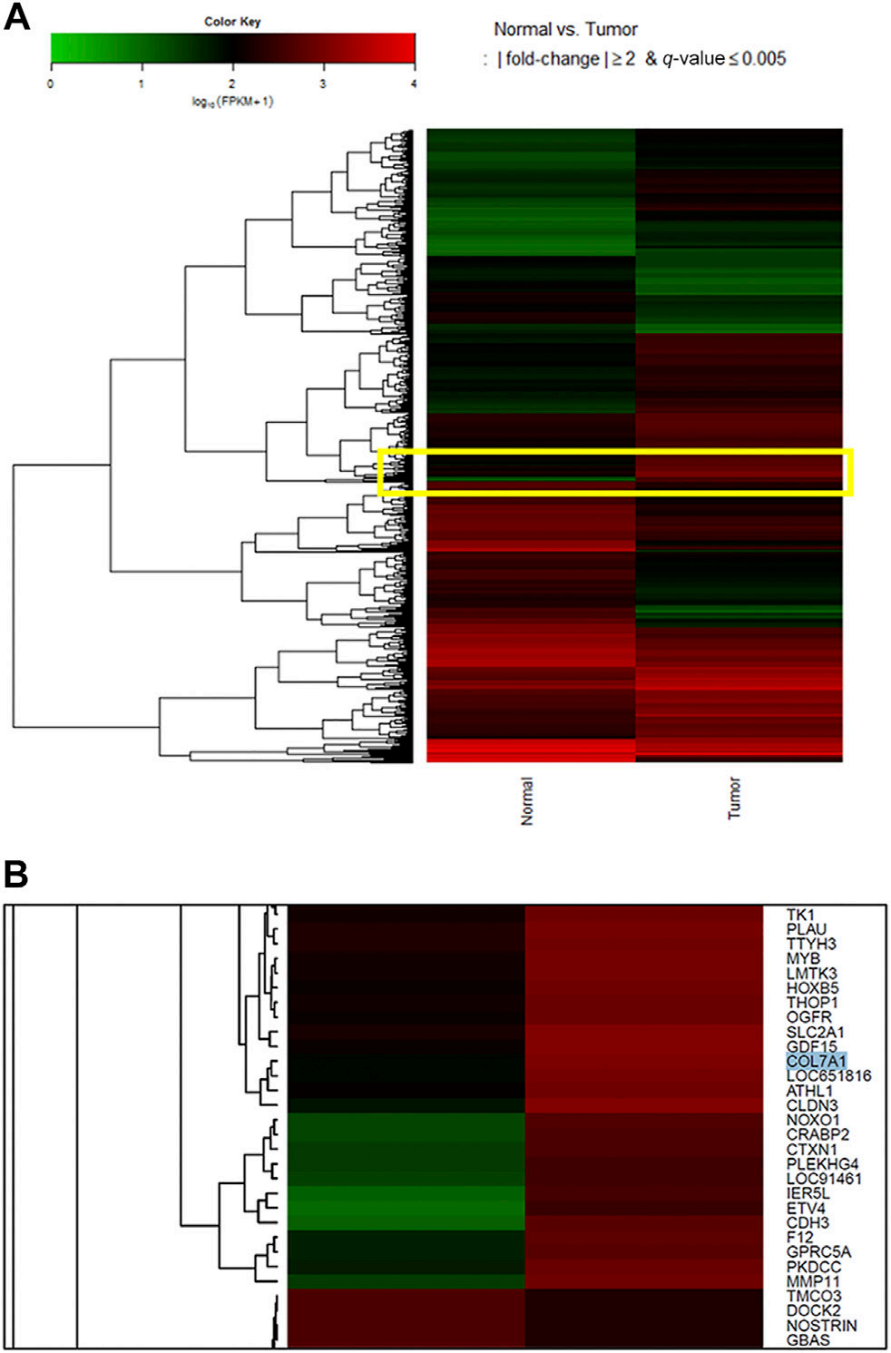
Heatmap for log_10_ (fragments per kilobase of transcripts per million mapped reads, FPKM) values to compare gene expression across genes and samples. After pairing and clustering cancerous and noncancerous tissues, 1,662 differentially expressed genes were selected. During the functional annotation analysis, seven genes (COL1A1, COL7A1, CPA2, CPA3, PGA3, PGA4, and PGA5) were mappable to a protein digestion and absorption pathway (KEGG pathway: hsa 04,974). Among them, COL7A1 and COL1A1 were upregulated, and the others were downregulated [COL7A1: log2 (FC) = 3.679, *p* < 0.05, *Q* < 0.005; COL1A1: log2 (FC) = 3.281, *p* < 0.05, *Q* < 0.005]. When we focused on the genes in the yellow box **(A)**, COL7A1 in cancer tissue was highly expressed in the heatmap **(B)**. As COL7A1 was highly expressed in tumor tissues than in normal gastric tissues, we selected the gene as the target gene of our study. As for the color key, a log_10_ (FPKM+1) value of 1 is shown black because it is mostly the peak value of the FPKM density plot. For this plot, a value of 1 is added to the FPKM to avoid zero-based errors. Red squares, high expression; green squares, low expression; black squares, no difference.

During the functional annotation analysis, seven genes (COL1A1, COL7A1, CPA2, CPA3, PGA3, PGA4, and PGA5) were mappable to a protein digestion and absorption pathway (KEGG pathway: hsa 04974). Among them, COL7A1 and COL1A1 were upregulated, and the others were downregulated [COL7A1: log2(FC) = 3.679, *p* < 0.05, *Q* < 0.005; COL1A1: log2(FC) = 3.281, *p* < 0.05, *Q* < 0.005]. When we visualized the gene expression result in a heatmap and magnified the portion in the yellow box (Figure 1A), COL7A1 was upregulated in tumor tissue (red) compared with normal tissue (black, Figure 1B). As COL7A1 was highly expressed in tumor tissues than in normal gastric tissues, we selected the gene as the target gene of our study.

### Immunohistochemistry of COL7A1 in Cancerous and Normal Tissue

**FIGURE 2:**
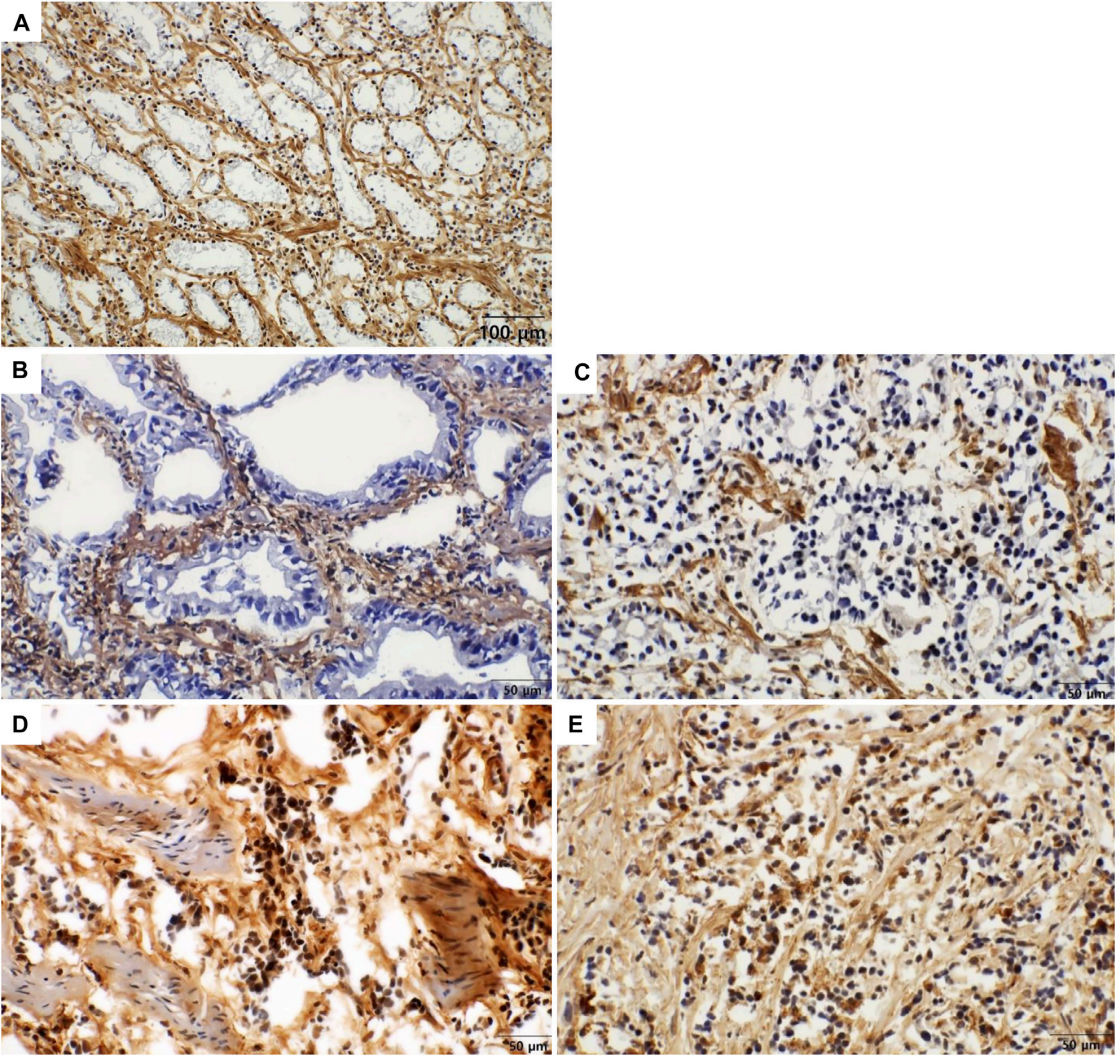
Immunohistochemical staining of COL7A1 in gastric cancer [**(A)** x200, **(B–E)** x400]. Representative images of intracellular and stromal expression of COL7A1. **(A)** In normal glandular cells show no expression of COL7A1 (intracellular score = 0). The stromal tissue surrounding the normal glands show strong expression of COL7A1 (extracellular score = 7). **(B)** In moderately differentiated gastric adenocarcinoma, tumor cells show no expression of COL7A1 in the cytoplasm (intracellular score = 0). The stromal tissue surrounding the cancer cells shows strong expression of COL7A1 (extracellular score = 7). **(C)** In poorly differentiated adenocarcinoma, tumor cells show no expression of COL7A1 in the cytoplasm (intracellular score = 0). The stromal tissue surrounding the cancer cells shows strong expression of COL7A1 (extracellular score = 7). **(D)** In poorly differentiated gastric adenocarcinoma, tumor cells show expression of COL7A1 in the nuclei and cytoplasm (intracellular score = 3). The stromal tissue surrounding the cancer cells show strong expression of COL7A1 (extracellular score = 7). **(E)** In poorly differentiated gastric adenocarcinoma, tumor cells show positivity in both nuclei and cytoplasm (intracellular score = 4. extracellular score = 7).

**FIGURE 3:**
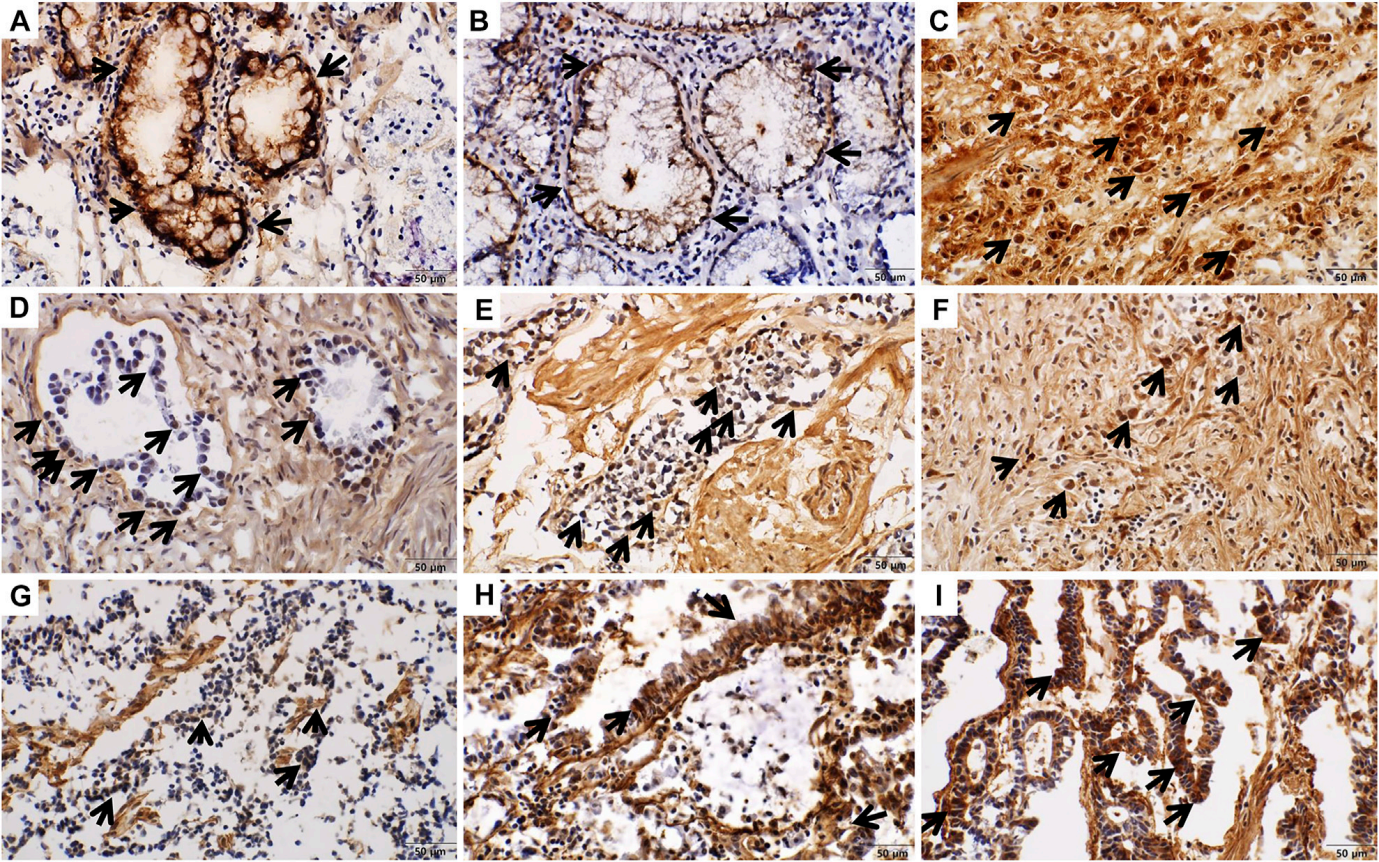
Intracellular immunohistochemical staining of COL7A1 (x400). Staining intensity was scored as 0 (negative), 1 (weakly positive), 2 (moderately positive), or 3 (strongly positive). **(A)** In normal gastric tissue, the goblet cells show strong expression of COL7A1 (intensity score = 3). **(B)** Non-specific membranous expression of COL7A1 was shown in normal gastric tissue. **(C)** Nuclear and cytoplasmic staining in gastric cancer tissue (intensity score = 3). **(D–F)** Nuclear staining of COL7A1 was shown in gastric cancer tissues (**(D)** intensity score = 1, **(E)** intensity score = 2, **(F)** intensity score = 3). **(G–I)** Cytoplasmic staining of COL7A1 was shown in gastric cancer tissues (**(G)** intensity score = 1, **(H)** intensity score = 2, **(I)** intensity score = 3).

The immunohistochemistry scores comparing cancer tissues (*n* = 200) with normal gastric tissues (*n* = 100) are shown in Table 1. The mean of total immunohistochemistry score of COL7A1 was higher in gastric cancer tissue than in normal stomach tissue (7.50 versus 6.93, *p =* 0.001). The proportion of tissues with a total immunohistochemistry score of more than eight was significantly higher in tumors than in normal tissues (28.5 versus 15.0%, *p* = 0.010). The score was higher in the stroma (extracellular) than in the nucleus or cytoplasm (intracellular) of both the main cancer cells and normal glandular cells (Table 1; Figure 2 and Figure 3).

**TABLE 1:** COL7A1 immunohistochemistry scores and site of expression from normal and tumor tissue.

	Normal (*n =* 100)	Tumor (*n =* 200)	*p* value
COL7A1 immunohistochemistry score	6.93 (6.30–7.56)	7.50 (7.22–7.78)	0.001
Total score			0.010^a^
≥8	15 (15.0)	57 (28.5)	
<8	85 (85.0)	143 (71.5)	
Intracellular	1.11 (0.58–1.63)	0.96 (0.73–1.19)	0.036
Extracellular	5.88 (5.47–6.29)	6.55 (6.36–6.73)	<0.001
COL7A1 site of expression			0.019^a^
Intracellular	20 (20.0)	66 (33.0)	
Extracellular	80 (80.0)	134 (67.0)	

All values are means (95% confidence interval) or number of tissues (percentages). The differences in scores between the normal and tumor tissue were calculated with the Mann-Whitney test except for

a χ^2^ test.

### Correlations Between COL7A1 Expression in Cancer Tissue and the Clinicopathological Features of Patients

The clinicopathologic characteristics of patients in the COL7A1-low and -high score groups are shown in Table 2. Fifty seven of the 200 patients had high COL7A1 scores (total score ≥ 8) in their cancer tissues (28.5%). The COL7A1-high group contained more female patients than the COL7A1-low group (47.4 versus 26.6%, *p =* 0.005). Cases in which the tumor location was the whole stomach were more frequently found in the COL7A1-high group than in the COL7A1-low group (17.5 versus 5.6%, *p =* 0.046). Among the twenty patients (20/200, 10%) diagnosed with distant metastases, twelve of them were in the COL7A1-high group (12/57, 21.1%), and eight were in the COL7A1-low group (8/143, 5.6%, *p =* 0.001).

**TABLE 2:** Comparison of clinicopathologic characteristics categorized by high/low immunohistochemistry scores and the expression site of COL7A1 in cancer tissues.

Characteristics	Total score			Site of expression		
	High (≥8) (*n =* 57)	Low (<8) (*n =* 143)	*p* value^b^	Intracellular (*n* = 66)	Extracellular (*n* = 134)	*p* value^b^
COL7A1 immunohistochemistry score	9.9 ± 1.6	6.7 ± 1.3	<0.001^d^	9.2 ± 2.4	6.7 ± 1.1	<0.001^d^
<8				9 (13.6)	134 (100.0)	<0.001
≥8				57 (86.4)	0 (0.0)	
COL7A1 site of expression			<0.001			
Extracellular	0 (0.0)	134 (93.7)				
Intracellular	57 (100.0)	9 (6.3)				
Age, yrs	62.8 ± 13.0	62.1 ± 12.5	0.697^d^	62.1 ± 14.0	62.5 ± 11.9	0.492^d^
Sex			0.005			0.015
Male	30 (52.6)	105 (73.4)		37 (56.1)	98 (73.1)	
Female	27 (47.4)	38 (26.6)		29 (43.9)	36 (26.9)	
Tumor location			0.046			0.054
Lower	28 (49.1)	70 (49.0)		32 (48.5)	66 (49.3)	
Middle	9 (15.8)	28 (19.6)		10 (15.2)	27 (20.1)	
Upper	10 (17.5)	37 (25.9)		13 (19.7)	34 (25.4)	
Whole	10 (17.5)	8 (5.6)		11 (16.7)	7 (5.2)	
Tumor size, cm	7.5 ± 2.8	7.4 ± 3.2	0.344^d^	7.6 ± 2.8	7.4 ± 3.2	0.117^d^
Gross type			0.478			0.256
Other	49 (86.0)	128 (89.5)		56 (84.8)	121 (90.3)	
Borrmann type IV	8 (14.0)	15 (10.5)		10 (15.2)	13 (9.7)	
Histologic type			0.452			0.494
Differentiated	16 (28.1)	48 (33.6)		19 (28.8)	45 (33.6)	
Undifferentiated	41 (71.9)	95 (66.4)		47 (71.2)	89 (66.4)	
Histologic subtype			0.110^c^			0.047^c^
Tubular	41 (71.9)	120 (83.9)		48 (72.7)	113 (84.3)	
Papillary	3 (5.3)	3 (2.1)		3 (4.5)	3 (2.2)	
SRC	8 (14.0)	8 (5.6)		10 (15.2)	6 (4.5)	
Mucinous	5 (8.8)	12 (8.4)		5 (7.6)	12 (9.0)	
Lauren			0.296			0.377
Intestinal	18 (31.6)	62 (43.4)		22 (33.3)	58 (43.3)	
Diffuse	27 (47.4)	58 (40.6)		32 (48.5)	53 (39.6)	
Other	12 (21.1)	23 (16.1)		12 (18.2)	23 (17.2)	
Depth of invasion			0.471			0.213
T2	6 (10.5)	25 (17.5)		6 (9.1)	25 (18.7)	
T3	22 (38.6)	51 (35.7)		26 (39.4)	47 (35.1)	
T4	29 (50.9)	67 (46.9)		34 (51.5)	62 (46.3)	
Number of metastatic LNs	11 ± 12	8 ± 7	0.110^d^	11 ± 12	8 ± 7	0.052^d^
LN metastasis			0.137			0.144
N0	10 (17.5)	18 (12.6)		11 (16.7)	17 (12.7)	
N1	4 (7.0)	25 (17.5)		5 (7.6)	24 (17.9)	
N2	9 (15.8)	31 (21.7)		11 (16.7)	29 (21.6)	
N3	34 (59.6)	69 (48.3)		39 (59.1)	64 (47.8)	
Distant metastasis			0.001			0.007
M0	45 (78.9)	135 (94.4)		54 (81.8)	126 (94.0)	
M1	12 (21.1)	8 (5.6)		12 (18.2)	8 (6.0)	
Pathologic stage^a^			0.017^c^			0.073^c^
I	3 (5.3)	8 (5.6)		3 (4.5)	8 (6.0)	
II	10 (17.5)	29 (20.3)		12 (18.2)	27 (20.1)	
III	32 (56.1)	98 (68.5)		39 (59.1)	91 (67.9)	
IV	12 (21.1)	8 (5.6)		12 (18.2)	8 (6.0)	
Lymphatic invasion			0.232			0.781
Absent	12 (21.1)	42 (29.4)		17 (25.8)	37 (27.6)	
Present	45 (78.9)	101 (70.6)		49 (74.2)	97 (72.4)	
Venous invasion			0.415			0.679
Absent	42 (73.7)	113 (79.0)		50 (75.8)	105 (78.4)	
Present	15 (26.3)	30 (21.0)		16 (24.2)	29 (21.6)	
Perineural invasion			0.667			0.849
Absent	24 (42.1)	65 (45.5)		30 (45.5)	59 (44.0)	
Present	33 (57.9)	78 (54.5)		36 (54.5)	75 (56.0)	

The COL7A1 immunohistochemistry scores, age, tumor size, and number of lymph nodes are presented as means ± SD. Values in parentheses are percentages.

a According to the eighth edition of the American Joint Committee on Cancer Classification.

b χ^2^ test except for

c Fisher’s exact test and

d Mann-Whitney test *LN* lymph node, *SRC* signet ring cell.

We compared the clinicopathologic characteristics according to the sites of COL7A1 expression (only extracellular expression versus extra- and intracellular expression), and the results are presented in Table 2. The total immunohistochemistry scores in the intracellular expression group were significantly higher than those in the extracellular-only expression group (9.2 ± 2.4 versus 6.7 ± 1.1, *p* < 0.001). Interestingly, the intracellular expression group contained a significantly high proportion of female patients and signet ring cell histologic subtype compared with the extracellular-only expression group (female 43.9 versus 26.9%, *p* = 0.015; signet ring cell 15.2 versus 4.5%, *p* = 0.047). The prevalence of distant metastasis was also significantly higher in the intracellular expression group than in the extracellular-only expression group (18.2 versus 6.0%, *p* = 0.007).

### COL7A1 Expression and Prognosis

**FIGURE 4:**
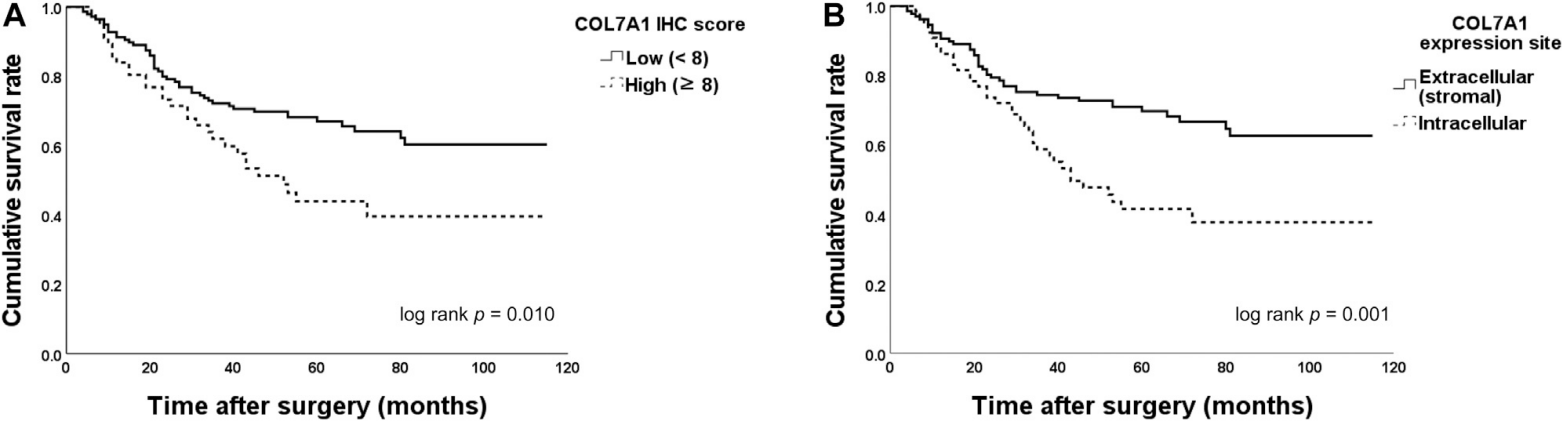
Overall survival (OS) between groups categorized by the type VII collagen (COL7A1) total immunohistochemistry score and site of expression. **(A)** When the patients were categorized by their immunohistochemistry scores (cut-off = 8), the OS of patients with high scores was significantly poorer than that of those with low scores (five-year OS 43.9% versus 66.9%, log-rank test *p =* 0.010). **(B)** The OS of patients with intracellular expression of COL7A1 was significantly lower than that of the patients with only extracellular (stromal) expression (five-year OS 41.5% versus 69.7%, log-rank test *p* = 0.001). IHC, immunohistochemistry.

Table 3 shows the results of the univariate and multivariate OS analyses. When patients were categorized to COL7A1-high and -low groups using the total immunohistochemistry score cut-off value (8), the OS between the groups differed significantly (≥8 versus <8, five-year OS 43.9% versus 66.9%, log-rank test *p =* 0.010, Figure 4A). When the patients were categorized by COL7A1 expression sites, the OS of the patients with intracellular expression was significantly poorer than that of the patients with only extracellular expression (five-year OS 41.5% versus 69.7%, log-rank test *p =* 0.001, Figure 4B). In the multivariate analysis (Table 3), the site of COL7A1 expression was an independent prognostic factor (hazard ratio 2.00, 95% confidence interval 1.26–3.16, *p =* 0.003), along with gross type (Borrmann type IV versus others, *p* = 0.001), T stage (*p* = 0.002), and lymphatic invasion (*p* = 0.025).

**TABLE 3:** Univariate and multivariate analyses of overall survival.

Characteristics	Univariate			Multivariate		
	HR	95% CI	*p* value	HR	95% CI	*p* value
COL7A1 immunohistochemistry score			0.011			0.121
≥8 versus <8	1.81	1.15–2.87				
COL7A1 site of expression			0.001			0.003
Intracellular versus Extracellular	2.07	1.32–3.25		2.00	1.26–3.16	
Age	0.99	0.98–1.01	0.403			
Sex			0.479			
Male versus Female	0.84	0.53–1.35				
Tumor location			<0.001			0.613
Middle versus Lower	1.17	0.61–2.24				
Upper versus Lower	1.46	0.82–2.59				
Whole versus Lower	4.44	2.38–8.29				
Tumor size, cm	1.08	1.02–1.14	0.006			0.732
Gross type			<0.001			0.001
Borrmann type IV versus Other	3.74	2.19–6.38		2.63	1.48–4.69	
Histologic type			0.442			
Undifferentiated versus Differentiated	1.21	0.74–1.97				
Lauren			0.009			0.904
Diffuse versus Intestinal	2.19	1.31–3.65				
Other versus Intestinal	1.45	0.73–2.86				
Depth of invasion			<0.001			0.002
T3 versus T2	1.91	0.63–5.69		1.35	0.45–4.08	
T4 versus T2	6.18	2.24–17.07		3.37	1.18–9.62	
LN metastasis			<0.001			0.079
N1 versus N0	0.58	0.19–1.76		0.58	0.18–1.82	
N2 versus N0	0.80	0.31–2.06		0.88	0.32–2.37	
N3 verssus N0	2.38	1.13–5.01		1.59	0.71–3.55	
Distant metastasis			<0.001			0.186
M1 versus M0	3.13	1.80–5.44				
Pathologic stage^a^			<0.001			0.815
II versus I	2.77	0.35–21.86				
III versus I	5.37	0.74–38.85				
IV versus I	13.91	1.84–105.06				
Lymphatic invasion			0.002			0.025
Present versus Absent	2.83	1.45–5.49		2.26	1.11–4.61	
Venous invasion			0.005			0.104
Present versus Absent	1.98	1.23–3.19				
Perineural invasion			0.002			0.351
Present versus Absent	2.19	1.33–3.59				

a According to the eighth edition of the American Joint Committee on Cancer Classification

*CI* confidence interval, *HR* hazard ratio, *LN* lymph node.

### Association Between COL7A1 Expression and Distant Metastasis

**FIGURE 5:**
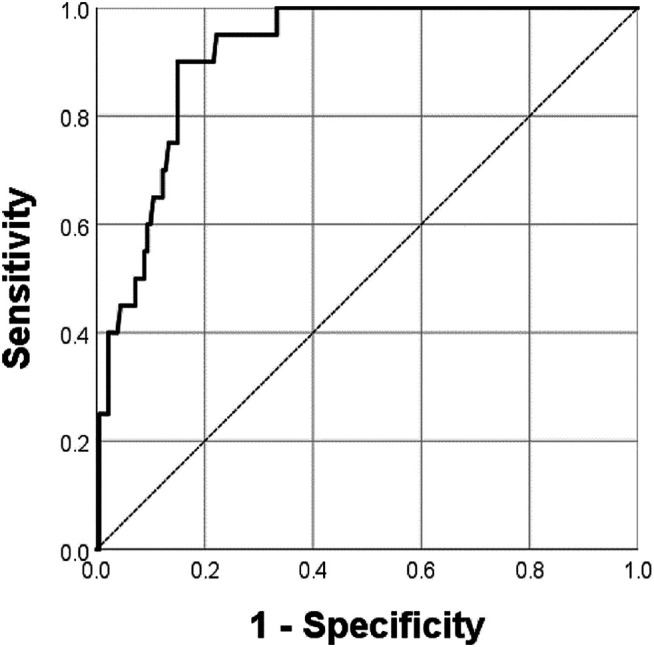
The receiver operator characteristic curve of the logistic regression model predicting distant metastases. The multinomial logistic regression model showed an AUC of 0.912 (*p <* 0.001, 95% confidence interval 0.866–0.959) in predicting distant metastases.

In the immunohistochemistry results, 20 patients had distant metastases, of whom 12 were in the COL7A1-high group. The peritoneum was the most common site (17/20, 85%), followed by distant lymph nodes (2/20, 10%) and the liver (1/20, 5.0%). The high score group showed a high prevalence of distant metastases, especially at the peritoneum (11/12, 91.7%). In the multinomial logistic regression for predicting distant metastases, the immunohistochemistry score (≥8 versus <8, odds ratio 4.45, 95% confidence interval 1.40–14.16, *p* = 0.011), serosa invasion depth (*p* = 0.024), number of metastatic lymph nodes (*p* = 0.002), and Lauren type (*p* = 0.031) were independent risk factors (Table 4). The regression model showed 91.5% predictive accuracy with an AUC of 0.912 (*p* < 0.001, 95% confidence interval 0.866–0.959, Figure 5) in predicting distant metastases.

**TABLE 4:** Multinomial logistic regression to predict distant metastasis (*n* = 20) using the clinicopathologic factors of patients whose cancer tissues were evaluated with COL7A1 immunohistochemistry (*n* = 200).

Variables	Odds ratio	95% confidence interval	*p* value
Immunohistochemistry score			0.011
≥8	4.45	1.40–14.16	
<8	1.00		
T stage			0.024
T4	5.62	1.26–25.14	
T2 and T3	1.00		
Metastatic lymph nodes (No.)	1.09	1.03–1.15	0.002
Lauren			0.031
Intestinal	1.00		
Diffuse	0.19	0.05–0.74	
Other	0.13	0.02–0.94	
Perineural invasion			0.080
Yes	4.43	0.84–23.47	
No	1.00		

The factors included in the first step of the model were sex, age, tumor location, gross type, histology type, Lauren type, tumor size, T stage, metastatic lymph nodes, lymphatic invasion, perineural invasion, venous invasion, total immunohistochemistry score, and the expression site of COL7A1.

The backward regression model was statistically significant (*p* < 0.001). The Nagelkerke R^2^ (R^2^_N_) was 0.418, and the *p* value was 0.695 in the Hosmer-Lemeshow test. The ninth step of this model predicted 176 patients without distant metastases and seven patients with distant metastases. Generally, this final model showed 91.5% predictive accuracy.

## Discussion

To our knowledge, we are the first to reveal the association between COL7A1 expression and gastric cancer through immunohistochemistry and to confirm its prognostic significance. COL7A1 expression was denser in cancer tissue than in normal tissue, and most of that expression was found in the extracellular matrix. This high expression score of COL7A1 was an independent risk factor for distant metastasis. Interestingly, patients with intracellular COL7A1 expression showed poorer OS than those with extracellular COL7A1 expression, and the site of expression was an independent prognostic factor for OS.

Using microarray technology, a previous gene expression analysis in gastric cancer found that the expression of several collagen genes increased in gastric cancer tissue, and COL7A1 was one of them (Cancer 119.1, Normal 46.7, Cancer/Normal 2.6, *p* < 0.05) [16]. In this study, we also began with a DNA microarray to explore the expression of various genes in gastric cancer and found that two types of collagen were highly expressed in cancer tissues: COL7A1 and COL1A1. Because COL1A1 is a common type of collagen and had been studied before [8], we chose COL7A1 for further investigation as a candidate prognostic biomarker. To investigate the distribution of the final target protein (COL7A1), which is a component of the tumor microenvironment, and determine its prognostic significance, we decided to use immunohistochemistry, which is an easy, cost effective, and widely available method that every pathology laboratory can perform [17].

Currently, an interesting theory is being investigated: that cancer is not only a problem of tumor cells but also a disease that causes a complicated, imbalanced environment surrounding the tumors. In this regard, cancer research has increasingly shifted to the tumor microenvironment. Among the many components of the tumor microenvironment, collagen provides the major structural framework of the extracellular matrix and can thus aid or restrain tumor progression [18].

In experiments with gastric cancer, fibroblasts around peripherally located tumor cells increased their collagen synthesis, reflecting a desmoplastic reaction to the cancer [19, 20]. Similar to previous studies, our immunohistochemistry results confirm that collagen synthesis and extracellular deposition increase in cancer tissue. Further research is needed to determine how cancer cells induce the fibroblasts to produce unusual types of collagen, especially type VII, and the mechanism by which type VII collagen contributes to cancer progression. Several explanations are possible. Increased tumor stromal collagen could block infiltrating cytotoxic immune cells and allow the tumor to escape from the host immune system in gastric cancer [21]. A second possibility is that, because type VII collagen functions as anchoring fibrils [7] between the cancer epithelium and the stroma, increased COL7A1 expression could contribute to cancer cell invasion.

Previous research investigated the clinical and pathophysiological associations between various types of collagen (though not type VII collagen) and gastric cancer. The previously mentioned study of type I collagen, one of the most widespread and abundant collagens, found that it could be a candidate prognostic factor in gastric cancer. Those researchers used real-time quantitative PCR to evaluate the expression of COL1A1 and COL1A2 in tissue. The mRNA expression was high in advanced cancer tissue, and having high expression correlated with low OS [8]. In scirrhous type gastric cancer, miR-143 is a critical mediator of collagen type III expression, which could support the progression of fibrillar formation [22]. Probably, increased collagen expression correlates with the scirrhous type of gastric cancer. Another study that performed a microarray meta-analysis found that type VI collagen (COL6A3) was regularly overexpressed in gastric cancer cells and suggested that gene could act as an oncogene [23].

Above all, we also found intracellular expression of COL7A1 in tumor cells. That phenomenon was especially frequent in female patients and signet ring cell type cancers. In addition, the total score for COL7A1 expression and prevalence of distant metastasis were also significantly higher in cancers with intracellular expression than in those with only extracellular expression. In the multivariate analysis, the site of COL7A1 expression (intra- or extracellular) was a significant prognostic factor for five-year OS. Signet ring cell type cancer frequently had intracellular expression of COL7A1, and cancers with intracellular expression showed high overall immunohistochemistry scores compared with cancers with only extracellular expression of COL7A1. The intracellular expression of this unusual type of collagen in gastric cancer might thus indicate the aggressiveness of the cancer itself, leading to poor patient prognosis. It seems like that the production of collagen in the intracellular space of cancer cells might be associated with a dedifferentiation process such as an epithelial-mesenchymal transformation.

The presence of distant metastasis was 91.5% predictable using the total expression score and other variables in a logistic regression analysis. Because collagen is mostly produced by fibroblasts, this result could support the findings of other studies regarding the role of gastric cancer–associated fibroblasts in cancer progression and metastasis [24, 25]. Furthermore, we have provided clinical evidence for further experiments about how this uncommon type of collagen triggers or contributes to distant metastasis, especially peritoneal dissemination.

COL7A1 might be not only a novel prognostic biomarker but also a therapeutic target, especially when it is intracellularly expressed. Because type VII collagen is mostly distributed in human skin, systemic therapy targeting type VII collagen should be avoided due to possible dermatologic complications. In this study, high expression of COL7A1 and distant metastasis, especially in the peritoneum, were strongly associated. Therefore, an intraperitoneal approach to target therapy in selected patients, such as females, those diagnosed with signet ring cell type cancer, and those with peritoneal metastasis, could be possible.

Although we used more tissues for staining when compared to other studies, the quality of staining might be poor as we could not use fresh tissues or formaldehyde-paraffin fixed tissues other than frozen tissues due to retrospective character of this study. Also, nuclear staining of COL7A1 needs functional explanation to clearly exclude nonspecific reactions. It remains unclear what precisely is showing antigenicity in the nuclei of tumor cells. Cross-reactivity to other antigen could be a plausible explanations for this observation. This may have happened during our experiment, however, we did not identify nuclear staining in normal tissues similar to that shown in cancer tissues with various intensities. Therefore, we analyzed the cases for general intracellular staining including both cytoplasmic and nuclear staining. Furthermore, to confirm the result of this study, we need more validation studies with tissues of good quality to reveal the biological interaction and mechanism between COL7A1 and tumor cells.

## Conclusion

In conclusion, we have shown that COL7A1 is overexpressed in gastric cancer and confirmed the clinical and prognostic significance of the intracellular expression of COL7A1. COL7A1 might be one of the tumor microenvironment components that contributes to cancer progression and distant metastasis. It could be a useful biomarker for diagnosis and a therapeutic target. Further investigations are needed to clarify the complex mechanisms of carcinogenesis and the interaction between COL7A1 and various components of the tumor microenvironment.

## Data Availability

The raw data supporting the conclusion of this article will be made available by the authors, without undue reservation.

## Abbreviations

AUC, area under the receiver operating characteristics curve; CI, Confidence interval; DAVID, Database for Annotation, Visualization, and Integrated Discovery; DEG, differentially expressed genes; FC, fold change; 5YOS, 5-years overall survival; HR, hazard ratio; KEGG, Kyoto Encyclopedia of Genes and Genomes; LN, lymph node; OS, Overall survival; RT, room temperature; SRC, signet ring cell.
